# Large-Scale Quantitative Proteomic Analysis during Different Stages of Somatic Embryogenesis in *Larix olgensis*

**DOI:** 10.3390/cimb45030130

**Published:** 2023-03-01

**Authors:** Jiayin Hou, Xuechun Wang, Weifeng Liu, Xiangning Jiang, Ying Gai

**Affiliations:** 1 College of Biological Science and Biotechnology, Beijing Forestry University, Beijing 100083, China; 2 National Engineering Research Center of Tree Breeding and Ecological Restoration, Beijing 100083, China; 3 The Tree and Ornamental Plant Breeding and Biotechnology Laboratory of National Forestry and Grassland Administration, Beijing 100083, China; 4 State Key Laboratory of Tree Genetics and Breeding, Beijing 100083, China

**Keywords:** somatic embryogenesis, quantitative proteomics, phytohormone, transcription factors, *Larix olgensis*

## Abstract

*Larix olgensis* is an economically important tree species native to northeastern China. The use of somatic embryogenesis (SE) is efficient and enables the rapid production of varieties with desirable qualities. Here, isobaric labeling via tandem mass tags was used to conduct a large-scale quantitative proteomic analysis of proteins in three critically important stages of SE in *L. olgensis*: the primary embryogenic callus, the single embryo, and the cotyledon embryo. We identified 6269 proteins, including 176 shared differentially expressed proteins across the three groups. Many of these proteins are involved in glycolipid metabolism, hormone response/signal transduction, cell synthesis and differentiation, and water transport; proteins involved in stress resistance and secondary metabolism, as well as transcription factors, play key regulatory roles in SE. The results of this study provide new insights into the key pathways and proteins involved in SE in *Larix*. Our findings have implications for the expression of totipotency, the preparation of synthetic seeds, and genetic transformation.

## 1. Introduction

Somatic embryogenesis (SE) is a process by which somatic cells differentiate into embryos that eventually give rise to entire plants. This process involves a series of morphological and biochemical changes that are regulated by several hormones [1]. In addition to providing a useful technique for genetic engineering, SE provides an excellent system for studying the development of embryos in higher plants [2]. Two key steps in the SE of gymnosperms (e.g., pine and fir) include the transition from the primary embryogenic callus (PEC) to the single embryo (SEM) and further development of the cotyledon embryo (CE). Clarification of molecular mechanisms underlying various SE steps can greatly enhance our understanding of plant development as well as related metabolic regulatory processes; such studies could also enable breeding of trees with enhanced wood quality [3].

Proteomic analyses have been used to identify proteins involved in SE and clarify the molecular mechanisms underlying SE in several plant species [4,5]. Many of the proteins that have been identified in these studies are involved in stress and detoxification, resistance to stress, the synthesis of hormones, signal transduction, the formation of cell walls, and lipid and energy metabolism. A few proteomic studies of SE in conifer species have been conducted. Proteomic analyses of SE in *Larix eurolepis* [6] and *Picea glauca* [7] have been conducted using two-dimensional gel electrophoresis coupled with mass spectrometry (MS). Proteomic analyses of SE in *Araucaria angustifolia* [8], *Picea balfouriana* [9], *Picea asperata* [10], *Picea abies* [11], and *Pseudotsuga menziesii* [12] have been conducted using a gel-free liquid chromatography platform. The expression of proteins involved in primary metabolism, phosphorylation, and redox regulation is up-regulated during the SE of *Larix principisrupprechtii* [13]. A whole proteome analysis of SE in *Pinus radiata* has revealed that proteins involved in sugar metabolism and translational regulation play key roles in the response to heat stress [14]. No studies to date have conducted a comprehensive analysis of the proteomic landscape in different developmental stages of SE in *Larix olgensis*, which is an economically important tree species native to China. Some of the main advantages of *L. olgensis* as a source of wood include its rapid early-stage growth, high resistance to insect pests, and ability to grow on barren land; its wood is also tough and resistant to corrosion and moisture.

Isobaric labeling by tandem mass tags (TMT) is a highly sensitive approach for analyzing changes in the expression of proteins under various physiological and environmental conditions [15]. Here, we identified differentially expressed proteins (DEPs) in three key developmental stages of SE (i.e., the primary embryogenic callus (PEC), the single embryo (SEM), and the cotyledon embryo (CE)) in *L. olgensis* using TMT-based proteomics and a liquid chromatography–tandem MS (LC–MS/MS). Our aim was to identify key proteins and pathways involved in SE in *L. olgensis*, in particular, transcription factors involved in hormone signal transduction and proteins related to stress resistance in secondary metabolic pathways. Our findings provided new insights into the roles of DEPs during SE in larches.

## 2. Materials and Methods

### 2.1. Plant Material and Sampling for Proteomic Analyses

*L. olgensis* seeds were collected from the Dagujia Tree Farm in Fushun City, Liaoning Province, China. Seeds in this experiment were cultured using the same culture medium as Gupta [16], according to the results of studies in our laboratory. In order to induce embryogenic callus, immature embryos were inoculated on medium supplemented with 1.0 mg/L 2,4-dichlorophenoxyacetic acid (2,4-D), 0.5 mg/L 6-benzylaminopurine (6-BA), and 0.3 mg/L kinetin (KT). After induction for 30 d, the embryogenic calli were then transferred to subculture medium supplemented with 0.1 mg/L 2,4-D, 0.05 mg/L 6-BA, and 0.03 mg/L KT and subcultured biweekly. After two subcultures, embryogenic calli were cultured for 10 d on maturation medium containing 50 g/L polyethylene glycol 4000, 20 mg/L ABA, and 10 mg/L silver nitrate. All procedures were conducted in the dark at 20–25 °C. The above three media comprise S medium [16] supplemented with 0.5 g/L glutamine and 0.5 g/L hydrolyzed casein. PEC samples included embryogenic calli subcultured more than twice but not placed on maturation medium. SEM samples included embryos that had been placed in maturation medium for 17–20 d. The CEs used for proteomic analysis included embryos cultivated for 30–35 d on maturation medium. Plant material was frozen immediately in liquid nitrogen after being collected and stored at −80 °C until testing. Each tested sample had a fresh weight greater than 0.5 g. Three biological replicate samples were collected for each stage of SE, and three technical replicates were performed for each sample in subsequent experiments.

### 2.2. Protein Extraction and Trypsin Digestion

The plant material collected during three different stages of SE was ground into powder in liquid nitrogen and dissolved in lysis buffer (8 M urea, 30 mM (4-(2-hydroxehtyl)-1-piperazineethanesulfonic acid, 1 mM phenylmethylsulfonyl fluoride, 2 mM ethylenediaminetetraacetic acid, and 10 mM dithiothreitol). The SDT lysis method was used for protein extraction [17]. Suspensions were sonicated for 10 min and centrifuged at 20,000× *g* for 30 min at 4 °C. Bradford assays were used to determine the protein concentration of the supernatant [18]. Proteins were trypsinized, and peptides were quantified by calculating the OD280 value following the methods of Winiewski [17].

### 2.3. TMT Labeling and High-pH Reversed-Phase Peptide Fractionation

To label peptides, the TMT labeling kit (Thermo Fisher Scientific, Waltham, MA, USA) was used by following the manufacturer’s instructions. A total of 100 μg of peptides were incubated in an acetonitrile solution containing TMT reagents for 2 h at room temperature; the mixture was then centrifuged, desalted, and dried. A high-pH reversed-phase peptide fractionation kit (Thermo Fisher Scientific, Waltham, MA, USA) was used to fractionate the labeled peptides. Briefly, a spin column containing pH-resistant reversed-phase resin was equilibrated with acetonitrile and 0.1% trifluoroacetic acid; the labeled peptide samples were then loaded, pure water was added, and the samples were centrifuged at low speed for desalting treatment. Finally, the column-bound peptides were subjected to gradient elution with increasing concentrations of high-pH acetonitrile solutions. After each eluted peptide sample was vacuum-dried, 0.1% formic acid (FA) was used to reconstitute lyophilized samples.

### 2.4. High-Performance Liquid Chromatography (HPLC) Fractionation Using Easy Nano-Liquid Chromatography (nLC) and LC–MS/MS Analysis

Samples were separated by HPLC using an easy nLC system (Thermo Fisher Scientific, Waltham, MA, USA); buffer A was 0.1% FA in water, and buffer B was water containing 0.1% FA and 84% acetonitrile. Solution A (95%) was used to equilibrate the chromatographic column, and samples were automatically loaded onto the loading column (Thermo Scientific Acclaim PepMap 100, 100 μm × 2 cm, nanoViper C18). Peptides were eluted at a flow rate of 300 nL/min with buffer B through an analytical column (Thermo Scientific EASY column, 10 cm, ID 75 μm, 3 μm, C18-A2).

A Q-Exactive mass spectrometer (Thermo Fisher Scientific, Waltham, MA, USA) was used to chromatographically separate and analyze samples with a precursor ion scan range of 300–1800 m/z and MS resolution of 70,000 at m/z 200. The AGC target value was set to 1e6. The maximum IT was set to 50 ms, and the dynamic exclusion duration was set to 60.0 s. The mass charge of peptides and peptide fragments was selected for MS/MS using the hollow cathode discharge mode with a normalized collision energy setting of 30 eV and an underfill ratio setting of 0.1%. The resolution for MS2 was set to 35,000 at m/z 200. Twenty MS2 scan fragments were acquired after each full scan.

### 2.5. Protein Identification and Quantitation

MASCOT 2.2 (Matrix Science, London, UK) and Proteome Discover 1.4 (Thermo Scientific, Waltham, MA, USA) software were used to quantitatively analyze raw MS-derived peptide data. Transcriptome data for *L. olgensis* previously collected by our research group were analyzed. The mass error tolerance was set to 20 ppm for peptide masses and 0.1 Da for fragmented ions; two missed cleavages were permitted in the trypsin digests. Carbamidomethyl, TMT10plex, and TMT10plex were set as fixed modifications, and oxidation and TMT10plex were set as variable modifications. Significant peptides were retained (FDR ≤ 0.05). Proteins were quantified using the median values of unique peptides and corrected using the median protein quantification values.

### 2.6. DEP Annotation and Functional Enrichment

DEPs were identified using the following criteria: |Fold change| > 1.2 and *p* < 0.05. GO analysis and KEGG pathway analysis were conducted on DEPs [19,20]. GO analysis involves the following four steps: sequence alignment, GO entry extraction, GO annotation, and annotation augmentation. First, the identified protein sequences were aligned with protein sequences in the NCBI non-redundant protein database using BLAST+ sequence alignment software. Second, the mapping function in the Blast2GO tool was used to extract GO terms according to the aligned sequences of all DEPs. Third, matching GO terms were annotated to relevant target proteins. Finally, the ANNEX module was used to further enhance the annotation information. KAAS software was used to assign KEGG pathways to target protein sequences, and KEGG mapper was used to retrieve KEGG orthology terms of homologous or similar proteins from relevant KEGG pathway databases [20]. The distributions of GO terms and KEGG pathways in the target protein set and the overall protein set were compared using Fisher’s Exact Test (*p* < 0.05).

### 2.7. RNA Extraction and RT-qPCR

To evaluate the consistency in the expression levels of proteins and the genes that encode them, RT-qPCR was conducted on 10 genes that were identified according to the proteomic data. EF1A1 was the only internal reference gene (Table S1). Primer Premier 6.0 software was used to design primers, and the primer sequences are listed in Table S1. A plant sample RNA extraction kit (Tiangen Biochemical Technology Co., Ltd., Beijing, China) was used to extract the total RNA of the samples. PrimeScript™ RT reagent kit with gDNA Eraser (TaKaRa) was used to reverse-transcribe cDNA. Experimental procedures were conducted following the manufacturers’ instructions. Total RNA (1 μg) was reverse-transcribed in each sample, and the final volume was 20 μL. RT-qPCR was conducted using an Mx3000p system (Agilent, Santa Clara, CA, USA) and SybrGreen qPCR Master Mix, 0.5 µL of forward and reverse primers, and 2 µL of template cDNA. We characterized the expression levels of 10 genes at three developmental stages in *L. olgensis*, and there were three biological replicates for each developmental stage. The expression levels of each gene in one sample were used as a standard for characterizing changes in expression levels in other samples; relative expression levels were quantified using the 2^−ΔΔCT^ method.

## 3. Results

### 3.1. SE in L. olgensis

**Figure 1:**
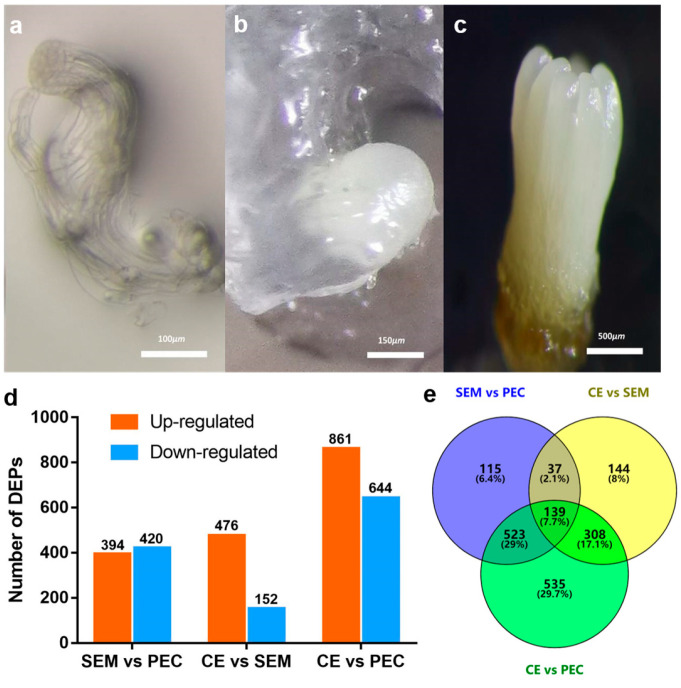
Images of three stages of SE. (**a**) primary embryogenic callus; (**b**) single embryo; (**c**) cotyledon embryo. DEPs in different comparison groups in SE. (**d**) Number of DEPs with up-regulated and down-regulated expression; (**e**) Venn diagram showing the distribution of DEPs shared and unique to the three comparison groups: SEM vs. PEC (blue circle), CE vs. SEM (yellow circle), and CE vs. PEC (green circle).

To identify the proteins involved in SE in *L. olgensis*, we used plant tissue from three different stages of SE, the PEC, SEM, and CE, and conducted TMT-based quantitative proteomics. The PEC was light white, transparent, and filamentous, and the structure of the embryonic stalk was visible (Figure 1a). Following water stress and abscisic acid (ABA) treatment, the callus changed from PEC to SEM, which involved various external changes, including the yellowing of the callus and the loss of transparency, an increase in the volume of the embryogenic stalk, the elongation of stalk cells, and the development of a long, opaque globular embryo (Figure 1b). In the CE stage, a cotyledon structure formed at the top of the embryo, and the hypocotyl elongated; eventually, a mature CE formed (Figure 1c).

### 3.2. Identification of Proteins in Different Stages of SE via TMT-Based Quantitative Proteomics and DEP Annotation

TMT-based quantitative proteomic analysis was used to identify proteins in three different stages of SE (PEC, SEM, and CE) in *L. olgensis*. Quality control validation of MS data, including the mass error, peptide ion score, peptide molecular weight, peptide count, and protein sequence coverage distributions, revealed that the results of our analysis were accurate and reliable (Figure S1). PCA showed good repeatability and significant differences between groups, PCA1 and PCA2, which could explain 78% and 13% of total variance, respectively (Figure S2). A total of 73,414 matched spectra were obtained. Of these spectra, 6269 proteins (Table S) and 37,432 peptides were identified, including 1801 unique DEPs (|Fold change| > 1.2, *p* < 0.05, and false discovery rate (FDR) ≤ 0.05) and 33,665 unique peptides. The number of significant DEPs in each comparison group is shown in Figure 1d. A total of 814 DEPs were identified in the SEM vs. PEC comparison, including 394 and 420 DEPs with up-regulated and down-regulated expression, respectively. A total of 1505 DEPs were identified in the PEC vs. CE comparison, including 861 and 644 DEPs with up-regulated and down-regulated expression, respectively. A total of 628 DEPs were identified in the SEM vs. CE comparison, including 476 and 152 DEPs with up-regulated and down-regulated expression, respectively. To analyze shared and specific proteins in the three stages of SE, Venn diagrams were used to visualize DEPs that were shared and unique among the three comparison groups. A total of 115, 144, and 535 DEPs were specifically expressed in the SEM vs. PEC, CE vs. SEM, and CE vs. PEC comparison groups, respectively; a total of 139 DEPs were shared among all three of these comparison groups (Figure 1e).

### 3.3. Analysis of the Functions of the Major DEPs Involved in SE

#### 3.3.1. Gene Ontology (GO) Analysis of DEPs

**Figure 2:**
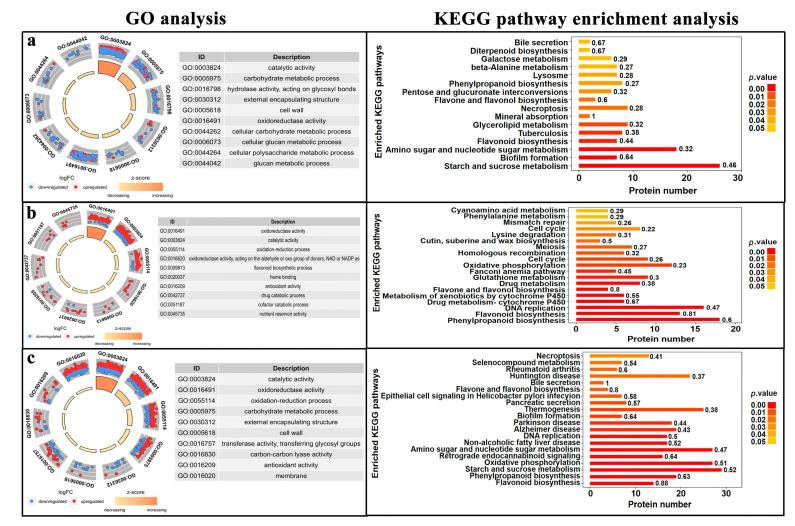
Results of the GO analysis and the KEGG pathway enrichment analysis of the (**a**) SEM vs. PEC, (**b**) CE vs. SEM, and (**c**) CE vs. PEC comparison groups.

GO analysis was used to assign GO terms to the DEPs in three categories: molecular function (MF), biological process (BP), and cellular component (CC) (Figure 2). The DEPs in the SEM vs. PEC comparison group were significantly enriched in 22 GO terms (FDR < 0.05), including nine MF terms, eight BP terms, and five CC terms. The DEPs in the CE vs. SEM comparison group were significantly enriched in 12 GO terms (FDR < 0.05), including six MF terms, two BP terms, and four CC terms. The DEPs in the CE vs. PEC comparison group were significantly enriched in 23 GO terms (FDR < 0.05), including 13 MF terms and 10 BP terms.

DEPs in the SEM vs. PEC comparison group were mostly enriched in the “catalytic activity” pathway (Figure 2a). DEPs in this comparison group were also enriched in the following pathways: the “carbohydrate metabolic process” pathway, which is involved in cell primary metabolism; the “cell wall” pathway, which is involved in the synthesis of cell structures; and the “transferase activity” and “hydrolase activity” pathways, which are involved in secondary metabolism. DEPs in the CE vs. SEM comparison group were enriched in oxidoreductase activity pathways (Figure 2b). DEPs in the CE vs. PEC comparison group were enriched in catalytically active pathways, and the DEPs involved in primary metabolism, secondary metabolism, and cell structure in the CE vs. PEC comparison group were similar to the DEPs in the PEC vs. SEM comparison group (Figure 2c). DEPs enriched in various pathways in the CE vs. SEM comparison group were also observed in the CE vs. PEC comparison group, especially DEPs involved in stress resistance.

The expression of most of the DEPs involved in the “catalytic activity” pathway was down-regulated in the PEC vs. SEM comparison group (Figure 2a); however, the expression of these DEPs was up-regulated in the CE vs. PEC comparison group (Figure 2b). The number of DEPs involved in catalytically active pathways with up-regulated and down-regulated expression was the same in the CE vs. SEM and CE vs. PEC comparison groups (Figure 2c). The number of DEPs enriched in the carbohydrate anabolic pathway significantly differed among the three groups; the expression of most DEPs in this pathway was decreased in the PEC vs. SEM comparison group (Figure 2a), and the expression of most DEPs was up-regulated in the CE vs. PEC comparison group (Figure 2b). The expression of most DEPs was up-regulated in the CE vs. SEM comparison group, and the expression of a few DEPs was down-regulated in this comparison group (Figure 2c). Hydrolases acting on glycosyl bonds were significantly differentially expressed in the PEC vs. SEM comparison group but not in the CE vs. PEC and CE vs. SEM comparison groups; hydrolases acting on glycosyl bonds were not highly active during the PEC stage (Figure 2a).

#### 3.3.2. Kyoto Encyclopedia of Genes and Genomes (KEGG) Analysis

KEGG pathway enrichment analysis was conducted on DEPs (*p* < 0.05). DEPs were enriched in a total of 16, 21, and 27 pathways in the SEM vs. PEC, CE vs. SEM, and CE vs. PEC comparison groups, respectively (Figure 2). In the SEM vs. PEC comparison group, the most enriched pathways included “starch and sucrose metabolism”, “glycerolipid metabolism”, and “phenylpropanoid biosynthesis”. In the CE vs. SEM comparison group, the most enriched pathways included “phenylpropane biosynthesis”, “flavonoid biosynthesis”, and “cell cycle”. In the CE vs. PEC comparison group, the most enriched pathways included “flavonoid biosynthesis”, “phenylpropane biosynthesis”, and “starch and sucrose metabolism”.

#### 3.3.3. Transcription Factors (TFs) Involved in SE

TFs are the key regulators of the processes underlying SE. To clarify the expression patterns of TFs, TFs involved in the SE of *L. olgensis* were predicted and classified. The TF families identified in the different developmental stages are shown in Figure S3. A total of 262 and 309 differentially expressed TFs were identified in the PEC vs. SEM and SEM vs. CE comparison groups, respectively; a total of 57 differentially expressed TFs were shared among the three comparable groups of SE. After the protein sequences were aligned against sequences in the National Center for Biotechnology Information (NCBI) database, significant differentially expressed TFs belonging to 18 TF families were identified, including TFs in the AP2 family, NAM family, and WOX family.

### 3.4. Hierarchical Cluster Analysis of DEPs at Different Stages of SE

**Figure 3:**
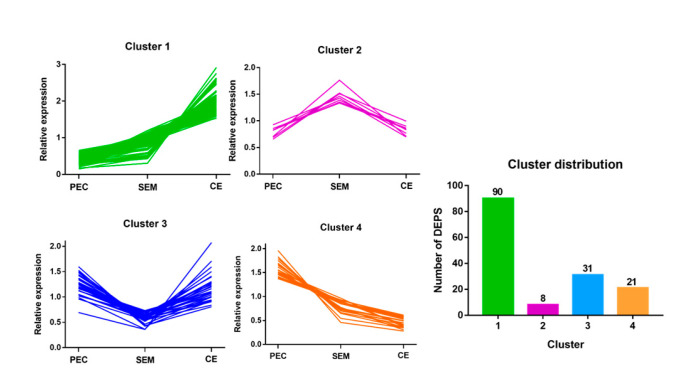
Hierarchical cluster analysis of DEPs. DEPs in PEC, SEM, and CE were classified into four clusters according to their expression patterns.

To characterize changes in the expression of proteins, hierarchical cluster analysis was conducted on the DEPs identified in PEC, SEM, and CE during SE in *L. olgensis*. DEPs were classified into four clusters according to their expression patterns, which included DEPs that were consistently up- regulated (cluster 1), DEPs that were initially up-regulated and then became down-regulated (cluster 2), DEPs that were initially down-regulated and then became up-regulated (cluster 3), and DEPs that were consistently down-regulated (cluster 4) (Figure 3 and Table S3). The expression of the 90 DEPs assigned to cluster 1 continuously increased throughout SE; these DEPs were mainly involved in “flavonoid biosynthesis”, including “Chalcone isomerase (CHI)”. The expression of the eight DEPs assigned to cluster 2 peaked from PEC to SEM and then decreased from SEM to CE; these DEPs were mainly involved in “diterpenoid biosynthesis”, including “Ent-kaurenoic acid oxidase 1”. The expression of the 31 DEPs assigned to cluster 3 decreased from PEC to SEM and then increased from SEM to CE; these DEPs were mainly involved in “phenylpropane biosynthesis”, “phenylalanine biosynthesis”, and “tyrosine and tryptophan biosynthesis”, including “Peroxidase 4”. The expression of the 21 DEPs assigned to cluster 4 decreased throughout SE; these DEPs were mainly involved in “glycolysis and gluconeogenesis” and “starch and sucrose metabolism”, including “Homeobox (WOX)”.

### 3.5. Correlation Analysis of Transcriptome and Proteome Data

#### 3.5.1. Correlations between Differentially Expressed Genes (DEGs) and DEPs

**Figure 4:**
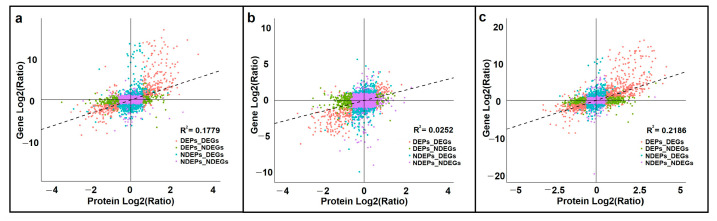
Correlations in the expression levels of proteins and associated transcripts for the (**a**) SEM vs. PEC, (**b**) CE vs. SEM, and (**c**) CE vs. PEC comparison groups. DEPs: differentially expressed proteins; DEGs: differentially expressed genes; NDEPs: no differentially expressed proteins; NDEGs: no differentially expressed genes.

An analysis of transcriptome–proteome correlations during SE in *L. olgensis* was conducted. Specifically, we characterized correlations between the number of quantifiable proteins or DEPs and their corresponding transcripts (Table S4). The expression patterns of transcripts were generally consistent with the expression patterns of proteins, as the transcripts corresponding to more than 99.6% of the quantifiable proteins were detected in the three comparison groups. In addition, the transcripts corresponding to 33.8%, 17.1%, and 29.9% of the significant DEPs were detected in the SEM vs. PEC, CE vs. SEM, and CE vs. PEC comparison groups, respectively (Table S4). The correlation coefficients between the expression levels of the quantifiable proteins and the expression levels of their corresponding transcripts were 0.42, 0.16, and 0.47 in the SEM vs. PEC, CE vs. SEM, and CE vs. PEC comparison groups, respectively (Figure 4). The correlation coefficients between the expression levels of DEPs and the expression levels of their corresponding DEGs were 0.83, 0.65, and 0.85, respectively (Figure 4).

#### 3.5.2. Analysis of the Relative Expression Levels of DEPs in the Proteome and DEGs in the Transcriptome by Quantitative Real-Time Polymerase Chain Reaction (RT-qPCR)

**Figure 5:**
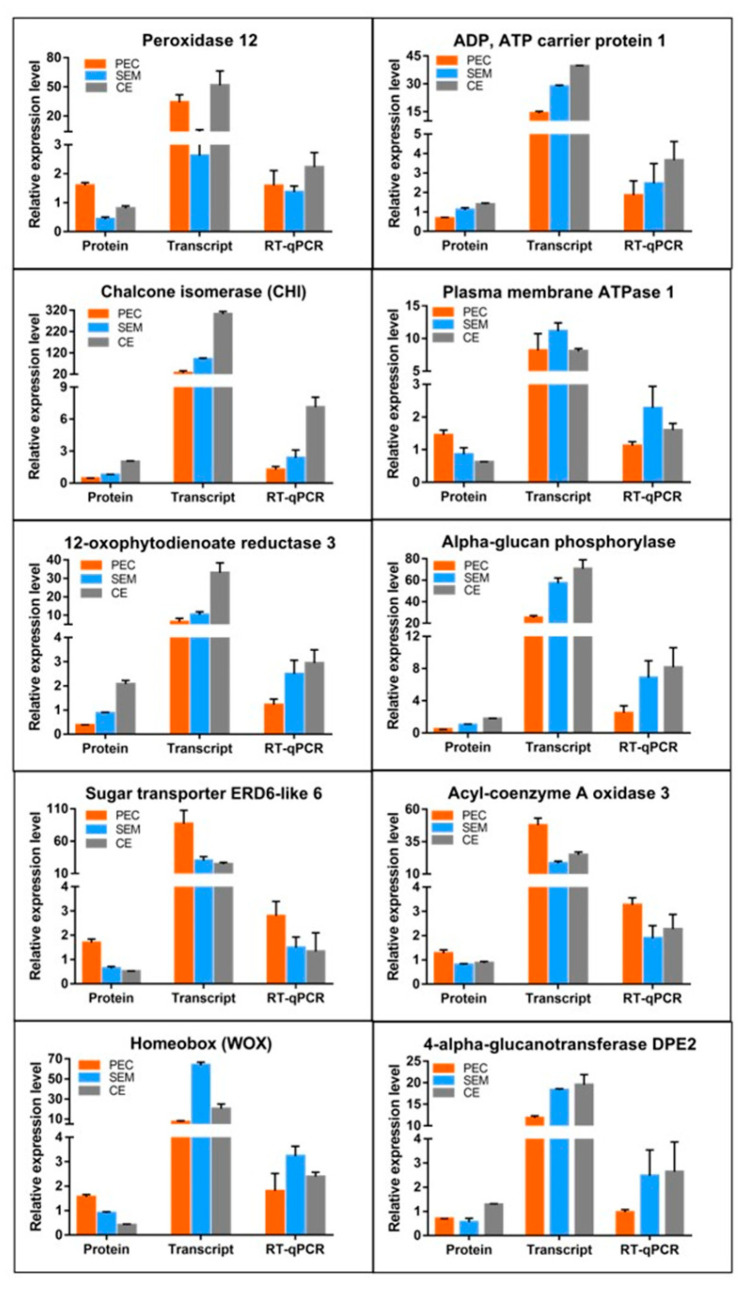
Relative expression levels of DEPs in the proteome and the expression of their corresponding transcripts in the transcriptome, according to RT-qPCR.

To verify that the expression levels of messenger RNAs (mRNAs) and DEPs were consistent, 10 candidate DEPs involved in growth and development processes in SE in *L. olgensis* were analyzed using RT-qPCR. Expression levels of proteins and their corresponding mRNAs were similar for the following proteins: peroxidase 12, CHI, 12-oxophytodienoate reductase 3, sugar transporter ERD6-like 6, ADP, ATP carrier protein 1, alpha-glucan phosphorylase, and acyl-coenzyme A oxidase 3 (Figure 5). However, levels of protein and mRNA expression were inconsistent for the following proteins: WOX, plasma membrane ATPase 1, and 4-alpha-glucanotransferase DPE2 (Figure 5).

## 4. Discussion

**Figure 6:**
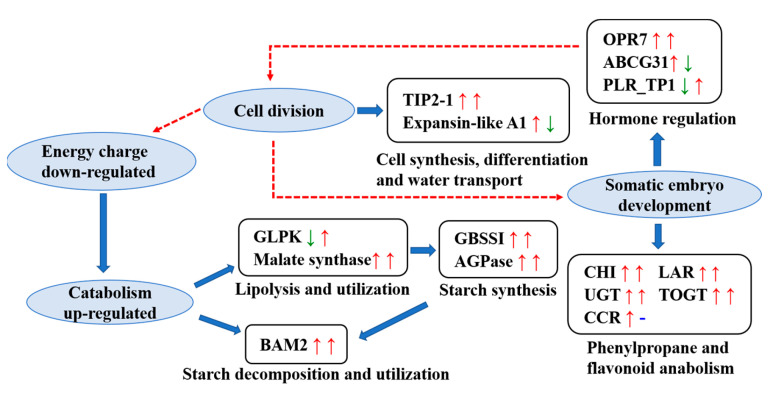
The key signaling pathways and proteins involved in SE in *L. olgensis*. The first arrow behind each protein represents its expression from PEC to SEM, and the second arrow represents its expression from SEM to CE. Up arrows indicate significant up-regulation; down arrows indicate significant down-regulation; and blue horizontal lines indicate neither up-regulation nor down-regulation. Dotted red lines indicate indirect action.

We used the TMT-based quantitative proteomic analysis to identify and characterize expression patterns of proteins involved in SE in *L. olgensis*. By using bioinformatic methods, we identified a total of 1801 DEPs and other various signaling pathways in which these DEPs participate. These DEPs were involved in the following signaling pathways: glycolipid metabolism, secondary metabolism such as phenylpropane and flavonoid biosynthesis, hormone signal transduction, and cell proliferation (Figure 6). We analyzed correlations between transcriptomic and proteomic data as well as the expression of ten candidate DEPs involved in the growth and development of *L. olgensis* in SE. Our findings provide comprehensive insights into the expression patterns of key proteins involved in SE in *L. olgensis*.

### 4.1. DEPs Involved in the Glucose and Lipid Metabolism Pathways

Glycolipid metabolism plays a key role in regulating the energy supply during embryonic development in plants, as it provides the carbohydrates required for the growth and development of the embryo and mediates the synthesis of functional polysaccharides, cellulose, and lipid plant hormones. The expression of malate synthase increased throughout SE in *L. olgensis*; the expression level of malate synthase at the end of the CE stage was nearly five times that observed in the PEC stage. This indicates high glyoxylate cycle activity; the products of fat oxidation and decomposition were converted into intermediate substances that could participate in gluconeogenesis. The expression of ADP-glucose pyrophosphorylase (AGPase), granule-bound starch synthase I (GBSSI), and beta-amylase 2 (BAM2) also increased significantly during SE. BAM2 mediates the decomposition of starch, and the increase in the expression of BAM2 during SE was greater than the increase in the expression of AGPase. This suggests that the demand for energy increases greatly during SE and that energy needs cannot be met via the consumption of stored fat. Consequently, stored starch needs to be broken down to meet energy needs. Glycerol kinase (GLPK) is a key enzyme in the first step of glycerol catabolism [21]. Glycerol is an important carbon source during seed germination and seedling development that can mediate the separation of water-soluble components and affect cell proliferation. In our study, the expression of GLPK decreased from PEC to SEM and increased from SEM to CE. GLPK might play a key role in cell proliferation in PEC and gluconeogenesis in CE; its role in gluconeogenesis might be particularly important. Previous studies have shown that GBSSI controls the synthesis of amylose in storage organs (e.g., the seeds and endosperm) in wheat, rice, and maize. TFs in the bZIP, AP2/EREBP, and NAC families regulate the expression of the gene encoding GBSSI [22,23]. Several TFs in these families were significantly differentially expressed in this study (Figure S3). Embryos with the nam mutation in Arabidopsis fail to develop shoot apical meristems, and the cotyledons are often laterally fused; NAM TFs are in the NAC family. A total of 12 TFs in the NAM subfamily were significantly differentially expressed in the PEC vs. SEM comparison group, and 15 TFs were significantly differentially expressed in the SEM vs. CE comparison group. These findings indicate that during SE in *L. olgensis*, NAM TFs regulate the expression of GBSSI and thus affect cotyledon formation.

From PEC to CE, several lipids are decomposed to produce compounds such as starch, and these reactions are catalyzed by enzymes involved in glucose and lipid metabolism. These compounds provide energy for the processes underlying rapid increases in the number of cells. The roles of different carbon sources and key enzymes mediating different carbon source synthesis pathways in SE vary, and embryoid bodies could be more efficiently generated via improvements in the provision of different carbon sources at different stages.

### 4.2. Main DEPs in Secondary Metabolic Pathways Involved in Stress Resistance

Flavonoids are an important class of secondary plant metabolites that play key physiological roles in plant growth, development, and stress resistance; the biosynthesis of flavonoids requires an interaction between multiple enzymes [24]. In flavonoid biosynthesis, CHI catalyzes the production of naringenin and mediates the synthesis of anthocyanins [25], which are converted into catechins by the anthocyanin reductase (LAR) [26]. Catechins play key roles in diverse processes, including immune regulation and antioxidant pathways. UDP glycosyltransferase (UGT) catalyzes the glycosylation of key proteins involved in the flavonoid biosynthesis pathway, which is a critically important step at the end of the flavonoid biosynthesis pathway. UGT79 has been shown to enhance resistance to salt stress and low-temperature tolerance in *Arabidopsis* [27]. Scopoletin is a key phenolic plant antitoxin that can be generated via glycosylation catalyzed by tobacco salicylic acid and pathogen-inducible UGT (TOGT) [28]. In our study, the expression levels of CHI, LAR, UGT, and TOGT increased significantly from PEC to SEM and from SEM to CE. This indicates that CHI, LAR, UGT, and TOGT play key roles in mediating salt stress resistance, low-temperature tolerance, and antitoxic activity during SE in *L. olgensis*. In the phenylpropane biosynthesis and phenylalanine metabolism pathways, cinnamoyl-CoA reductase (CCR) is the enzyme that catalyzes the first rate-limiting step of lignin synthesis, which enhances the toughness of the cell wall and the resistance of plants to abiotic stress [29]. The expression level of CCR increased from PEC to SEM, but no change in the expression level of CCR was observed from SEM to CE. CCR might play a key role in mediating the resistance of *L. olgensis* to stress by regulating the content of lignin from PEC to SEM. Overall, the expression of secondary metabolism enzymes was enhanced during SE in *L. olgensis*.

### 4.3. Major Regulatory DEPs Involved in Phytohormone Signal Transduction and Cell Proliferation

Hormones play a key regulatory role in the development of somatic plant embryos. The rate of somatic embryo production can be increased by modifying the contents of different hormones. The 12-oxophytodienoate reductase 7 (OPR7) plays a role in JA biosynthesis in maize [30]. The expression of the TF AP2/ERF1 in *Arabidopsis* is induced by JA [31], and AP2 has been shown to be involved in the development of seeds in *Arabidopsis* [32]. In our study, the expression of OPR7 increased from PEC to SEM and from SEM to CE, and the expression of several AP2 family-related proteins was detected (Figure S3). The expression level of JA increased as the OPR7 content increased, and this increased AP2 activity during SE in *L. olgensis*, which promoted the dedifferentiation and rapid proliferation of embryonic cells. ABA is a seed germination inhibitor, and ABC transporter G subfamily member 31 (ABCG31) mediates the transport of ABA from the endosperm to the embryo [33], which inhibits the development of the embryo under adverse environmental conditions. The content of ABCG31 increased from PEC to SEM, and this promoted the release of more ABA into somatic embryos, which inhibited embryo development. However, the content of ABCG31 decreased from SEM to CE to a level that was slightly higher than that observed in PEC, and this weakened the inhibition of embryonic development. Bifunctional pinoresinol-larch resinol reductase 1 (PLR_Tp1) is a key enzyme involved in lignan synthesis in plants. Lignans play key roles in stress resistance and sex determination in plants; they also regulate the growth of plants [34]. In our study, the content of PLR_Tp1 decreased from PEC to SEM, and no changes in the content of PLR_Tp1 were observed from SEM to CE. Thus, the content of PLR_Tp1 and sex determination hormones might remain low in *L. olgensis* to prevent the differentiation of somatic embryos.

Proteins that are involved in cell proliferation and browning play key roles in embryo growth and development. Expansin-like A1 is a regulatory protein in the cell wall that can break the hydrogen bond separating hemicellulose and cellulose, which causes the loosening and extension of the cell wall [35]. In this study, the expression of expansin-like A1 significantly increased from PEC to SEM, and this likely mediated the rapid proliferation of cells during the early embryonic development of *L. olgensis*. Tonoplast intrinsic protein (TIP) regulates the rehydration of protein storage vesicles in the early stage of seed germination as well as the fluidity of the vesicle membrane and the composition of the lipid membrane in the late stage of seed germination [36]. In our study, the expression of TIP2-1 significantly increased from PEC to SEM and from SEM to CE, and the final content of TIP2-1 in CE was nearly seven times that in PEC. TIP2-1 is an intrinsic vacuolar membrane protein that controls the flow of water into and out of cells. We hypothesized that TIP also plays a role in maintaining osmotic pressure during seed germination to prevent desiccation and abortion during SE in *L. olgensis*. UDP-glucosedehydrogenase (UGDH) catalyzes the rate-limiting step in cell wall polysaccharide synthesis; it controls the synthesis of cellulose and pectin [37], which is necessary for the syncytium to produce cell walls, and a lack of UGDH3 causes the syncytium to decrease in size [38]. In our study, the expression of UGDH3 was highest in PEC and gradually decreased from PEC to CE. We hypothesize that UGDH mainly affected cell wall synthesis in the early stages of SE in *L. olgensis*, but the effects of UGDH’s decrease in the middle and late stages of SE require further investigation. Peroxidase has been shown to catalyze the browning of plant embryonic tissue, which reduces the proliferation of embryonic cells [39]. In our study, the expression levels of most peroxidases significantly decreased from PEC to SEM and significantly increased from SEM to CE. The significant decrease in the expression of peroxidases from PEC to SEM indicates that peroxidase activity was weak during this period; this prevented the browning of the callus and promoted embryonic differentiation.

Overall, the expression of proteins involved in cell enlargement and propagation increased from PEC to CE. Furthermore, the expression of proteins involved in cell differentiation and tissue and organ construction remained low during SE. The maintenance of dedifferentiation and rapid growth are key characteristics of SE in *L. olgensis*.

### 4.4. TF-Related Proteins Involved in SE

Proteins in the WOX subfamily, which belongs to the Homeobox family, play key roles in regulating meristem development and embryonic development. Several studies have shown that WOX3 regulates tissue specificity and meristem evolution [40], and WOX4 and WOX5 regulate root development [41,42]. WOX8 promotes the formation of the cotyledon boundary, and WOX9 regulates the growth and maintenance of the meristem in *Arabidopsis* [43,44]. In our study, proteins related to the above TFs were involved in the regulation of SE. WUSCHEL-related homeobox 9 was a DEP, and its expression was highest in PEC; the expression of WUSCHEL-related homeobox 9 significantly decreased from PEC to SEM and from SEM to CE. These findings indicate that WOX-related proteins are involved in the initiation of somatic embryogenesis. It’s worth noting that levels of protein and mRNA expression were inconsistent for WOX in SE (Figure 5), suggesting that its protein levels may not only depend on the transcript level but also on the level of post-translation.

## 5. Conclusions

Our study is the first to utilize TMT-based proteomic techniques for identifying the key regulatory proteins involved in the SE of *L. olgensis*. During SE, the activities of enzymes involved in energy supply metabolism and secondary metabolism and stress-related enzymes (e.g., UGT and TOGT) increased. The activity of enzymes involved in hormone regulation and signal transduction (e.g., OPR7 and ABCG31) was altered to promote cell division. Expansin-like A1, UGDH, and TIP play key roles in regulating cell synthesis and differentiation and water transport. Several TFs (e.g., TFs in the NAM, WOX, and AP2 families) are involved in somatic embryo development in *L. olgensis* by regulating the content of hormones and the synthesis of energy metabolism-related enzymes. Additional experimental research is needed to clarify the regulatory effects of decreases in the UGDH content in the middle and late stages of SE. The aforementioned proteins play key roles in promoting the initiation of embryogenic cells, the proliferation of somatic embryos, and the regeneration of plants. Future studies are needed to clarify the potential functions of the genes, proteins, and pathways underlying the regulation of embryogenesis in *L. olgensis* identified in our study.

## Data Availability

The data presented in this study are available in the article and Sup-plementary Materials. The mass spectrometry proteomic data have been deposited to the ProteomeXchange Consortium (http://proteomecentral.proteomexchange.org (accessed on 27 February 2023)) via the iProX partner repository [45] with the dataset identifier PXD040378.

## Supplementary Materials

The following supporting information can be downloaded at: https://www.mdpi.com/article/10.3390/cimb45030130/s1. Figure S1: Quality control validation of the MS data; Figure S2: PCA plots from the proteomics at three stages of SE; Figure S3: TFs identified in different stages of SE in L. olgensis; Table S1: List of primers used for RT-qPCR; Table S2: Details of the identified proteins; Table S3: Details of the hierarchical cluster analysis of DEPs; Table S4: Correlation analyses between transcripts and quantifiable proteins or DEPs in the SEM vs. PEC, CE vs. SEM, and CE vs. PEC comparison groups.

## References

[1] Zimmerman J.L. (1993). Somatic embryogenesis: A model for early development in higher plants. Plant Cell.

[2] Yang X., Zhang X. (2010). Regulation of somatic embryogenesis in higher plants. Crit. Rev. Plant Sci..

[3] Guan Y., Li S.G., Fan X.F., Su Z.H. (2016). Application of somatic embryogenesis in woody plants. Front. Plant Sci..

[4] Zhu H.G., Cheng W.H., Tian W.G., Li Y.J., Liu F., Xue F., Zhu Q.H., Sun Y.Q., Sun J. (2018). iTRAQ-based comparative proteomic analysis provides insights into somatic embryogenesis in *Gossypium hirsutum* L.. Plant Mol. Biol..

[5] Liu B., Shan X., Wu Y., Su S., Li S., Liu H., Han J., Yuan Y. (2018). iTRAQ-Based quantitative proteomic analysis of embryogenic and non-embryogenic calli derived from a maize (*Zea mays* L.) inbred line Y. Int. J. Mol. Sci..

[6] Teyssier C., Maury S., Beaufour M., Grondin C., Delaunay A., Le Metté C., Ader K., Cadene M., Label P., Lelu-Walter M.A. (2014). In search of markers for somatic embryo maturation in hybrid larch (Larix × eurolepis): Global DNA methylation and proteomic analyses. Physiol. Plant..

[7] Lippert D., Zhuang J., Ralph S., Ellis D.E., Gilbert M., Olafson R., Ritland K., Ellis B., Douglas C.J., Bohlmann J. (2005). Proteome analysis of early somatic embryogenesis in Picea glauca. Proteomics.

[8] Fraga H.P.F., Vieira L.N., Heringer A.S., Puttkammer C.C., Silveira V., Guerra M.P. (2016). DNA methylation and proteome profiles of *Araucaria angustifolia* (Bertol.) Kuntze embryogenic cultures as affected by plant growth regulators supplementation. Plant Cell Tiss. Organ. Cult..

[9] Li Q., Zhang S., Wang J. (2015). Transcriptomic and proteomic analyses of embryogenic tissues in *Picea balfouriana* treated with 6-benzylaminopurine. Physiol. Plant..

[10] Jing D., Zhang J., Xia Y., Kong L., Ouyang F., Zhang S., Zhang H., Wang J. (2017). Proteomic analysis of stress-related proteins and metabolic pathways in *Picea asperata* somatic embryos during partial desiccation. Plant Biotechnol. J..

[11] Eliášová K., Konrádová H., Dobrev P.I., Motyka V., Lomenech A.M., Fischerová L., Lelu-Walter M.A., Vondráková Z., Teyssier C. (2022). Desiccation as a post-maturation treatment helps complete maturation of Norway spruce somatic embryos: Carbohydrates, phytohormones and proteomic status. Front. Plant Sci..

[12] Gautier F., Eliášová K., Leplé J.C., Vondráková Z., Lomenech A.M., Le Metté C., Label P., Costa G., Trontin J.F., Teyssier C. (2018). Repetitive somatic embryogenesis induced cytological and proteomic changes in embryogenic lines of *Pseudotsuga menziesii* [Mirb.]. BMC Plant Biol..

[13] Zhao J., Li H., Fu S., Chen B., Sun W., Zhang J., Zhang J. (2015). An iTRAQ-based proteomics approach to clarify the molecular physiology of somatic embryo development in Prince Rupprecht’s larch (*Larix principis-rupprechtii* Mayr). PLoS ONE.

[14] Castander-Olarieta A., Pereira C., Montalbán I.A., Mendes V.M., Correia S., Suárez-Álvarez S., Manadas B., Canhoto J., Moncaleán P. (2021). Proteome-wide analysis of heat-stress in *Pinus radiata* somatic embryos reveals a combined response of sugar metabolism and translational regulation mechanisms. Front. Plant Sci..

[15] Pagel O., Loroch S., Sickmann A., Zahedi R.P. (2015). Current strategies and findings in clinically relevant post-translational modification-specific proteomics. Expert Rev. Proteom..

[16] Gupta P.K., Durzan D.J. (1987). Biotechnology of somatic polyembryogenesis and plantlet regeneration in loblolly pine. Nat Biotechnol..

[17] Winiewski J.R., Zougman A., Nagaraj N., Mann M. (2009). Universal sample preparation method for proteome analysis. Nat. Methods.

[18] Bradford M.M. (1976). A rapid and sensitive method for the quantitation of microgram quantities of protein utilizing the principle of protein-dye binding. Anal. Biochem..

[19] Ashburner M., Ball C.A., Blake J.A., Botstein D., Butler H., Cherry J.M., Davis A.P., Dolinski K., Dwight S.S., Eppig J.T. (2000). Gene ontology: Tool for the unification of biology. The Gene Ontology Consortium. Nat. Genet..

[20] Kanehisa M., Goto S., Sato Y., Furumichi M., Tanabe M. (2012). KEGG for integration and interpretation of large-scale molecular data sets. Nucleic Acids Res..

[21] Tang Y., Shi Y., Jia B., Zhang Y., Wang Q. (2023). Evolution and function analysis of glycerol kinase GlpK in Pseudomonasaeruginosa. Biochem. Biophys. Res. Commun..

[22] Vrinten P.L., Nakamura T. (2000). Wheat granule-bound starch synthase I and II are encoded by separate genes that are expressed in different tissues. Plant Physiol..

[23] Cao R., Zhao S., Jiao G., Duan Y., Ma L., Dong N., Lu F., Zhu M., Shao G., Hu S. (2022). OPAQUE3, encoding a transmembrane bZIP transcription factor, regulates endosperm storage protein and starch biosynthesis in rice. Plant Physiol..

[24] Zhao Y., Zhou G., Sun T., Wang L., Xu Q., Liu J., Gao B. (2023). Metabolites and Plant Hormones Related to the Resistance Response to Feeding Stimulation and Leaf Clipping Control in Chinese Pine (*Pinus tabuliformis* Carr.). Curr. Issues Mol. Biol..

[25] Yu S., Li J., Peng T., Ni S., Feng Y., Wang Q., Wang M., Chu X., Fan Z., Li X. (2022). Identification of Chalcone Isomerase Family Genes and Roles of CnCHI4 in Flavonoid Metabolism in *Camellia nitidissima*. Biomolecules.

[26] Cheng J., Yu K., Shi Y., Wang J., Duan C. (2021). Transcription Factor VviMYB86 Oppositely Regulates Proanthocyanidin and Anthocyanin Biosynthesis in Grape Berries. Front. Plant Sci..

[27] Li P., Li Y.J., Zhang F.J., Zhang G.Z., Jiang X.Y., Yu H.M., Hou B.K. (2017). The Arabidopsis UDP-glycosyltransferases UGT79B2 and UGT79B3, contribute to cold, salt and drought stress tolerance via modulating anthocyanin accumulation. Plant J..

[28] Chong J., Baltz R., Schmitt C., Beffa R., Fritig B., Saindrenan P. (2002). Downregulation of a pathogen-responsive tobacco UDP-Glc:phenylpropanoid glucosyltransferase reduces scopoletin glucoside accumulation, enhances oxidative stress, and weakens virus resistance. Plant Cell.

[29] Liu D., Wu J., Lin L., Li P., Li S., Wang Y., Li J., Sun Q., Liang J., Wang Y. (2021). Overexpression of Cinnamoyl-CoA reductase 2 in Brassica napus increases resistance to Sclerotinia sclerotiorum by affecting lignin biosynthesis. Front. Plant Sci..

[30] Yan Y., Christensen S., Isakeit T., Engelberth J., Meeley R., Hayward A., Emery R.J.N., Kolomiets M.V. (2012). Disruption of OPR7 and OPR8 reveals the versatile functions of jasmonic acid in maize development and defense. Plant Cell.

[31] Lorenzo O., Piqueras R., Sánchez-Serrano J.J., Solano R. (2003). Ethylene Response Factor1 integrates signals from ethylene and jasmonate pathways in plant defense. Plant Cell.

[32] Jofuku K.D., Den Boer B.G., Van Montagu M., Okamuro J.K. (1994). Control of Arabidopsis flower and seed development by the homeotic gene APETALA. Plant Cell.

[33] Borghi L., Kang J., Ko D., Lee Y., Martinoia E. (2015). The role of ABCG-type ABC transporters in phytohormone transport. Biochem. Soc. Trans..

[34] Markulin L., Corbin C., Renouard S., Drouet S., Gutierrez L., Mateljak I., Auguin D., Hano C., Fuss E., Lainé E. (2019). Pinoresinol-lariciresinol reductases, key to the lignan synthesis in plants. Planta.

[35] Cosgrove D.J. (2000). Loosening of plant cell walls by expansins. Nature.

[36] Maurel C., Kado R.T., Guern J., Chrispeels M.J. (1995). Phosphorylation regulates the water channel activity of the seed-specific aquaporin alpha-TIP. EMBO J..

[37] Mason P.J., Hoang N.V., Botha F.C., Furtado A., Marquardt A., Henry R.J. (2023). Organ-specific expression of genes associated with the UDP-glucose metabolism in sugarcane (*Saccharum* spp. hybrids). BMC Genom..

[38] Siddique S., Sobczak M., Tenhaken R., Grundler F.M.W., Bohlmann H. (2012). Cell wall ingrowths in nematode induced syncytia require UGD2 and UGD. PLoS ONE.

[39] Abohatem M., Zouine J., Hadrami I.E. (2011). Low concentrations of BAP and high rate of subcultures improve the establishment and multiplication of somatic embryos in date palm suspension cultures by limiting oxidative browning associated with high levels of total phenols and peroxidase activities. Sci. Hortic..

[40] Zhang Z., Runions A., Mentink R.A., Kierzkowski D., Karady M., Hashemi B., Huijser P., Strauss S., Gan X., Ljung K. (2020). A WOX/Auxin Biosynthesis Module Controls Growth to Shape Leaf Form. Curr. Biol..

[41] Ji J., Strable J., Shimizu R., Koenig D., Sinha N., Scanlon M.J. (2010). WOX4 promotes procambial development. Plant Physiol..

[42] Oshchepkova E.A., Omelyanchuk N.A., Savina M.S., Pasternak T., Kolchanov N.A., Zemlyanskaya E.V. (2017). Systems biology analysis of the WOX5 gene and its functions in the root stem cell niche. Russ. J. Genet..

[43] Ueda M., Zhang Z., Laux T. (2011). Transcriptional activation of *Arabidopsis* axis patterning genes WOX8/9 links zygote polarity to embryo development. Dev. Cell.

[44] Wu X., Dabi T., Weigel D. (2005). Requirement of homeobox gene STIMPY/WOX9 for Arabidopsis meristem growth and maintenance. Curr. Biol..

[45] Chen T., Ma J., Liu Y., Chen Z., Xiao N., Lu Y., Fu Y., Yang C., Li M., Wu S. (2022). iProX in 2021: Connecting proteomics data sharing with big data. Nucleic Acids Res..

