# Supplementary material for: Large-Scale Quantitative Proteomic Analysis during Different Stages of Somatic Embryogenesis in Larix olgensis
Source: Curr Issues Mol Biol. 2023 Mar 1;45(3):2021–34. doi: 10.3390/cimb45030130 (PMC10047913; doi:10.3390/cimb45030130)
Supplement: Supplementary file 1 [file cimb-45-00130-s001.zip › Supplementary material-Figure.pdf]

Supplementary data (Figure)

Description

1. Quality control validation of Mass Spectrometer (MS) date was shown in Figure S1.
2. Principal component analysis (PCA) was shown in Figure S2.
3. The TF families identified in the different developmental stages are shown in Figure S3.

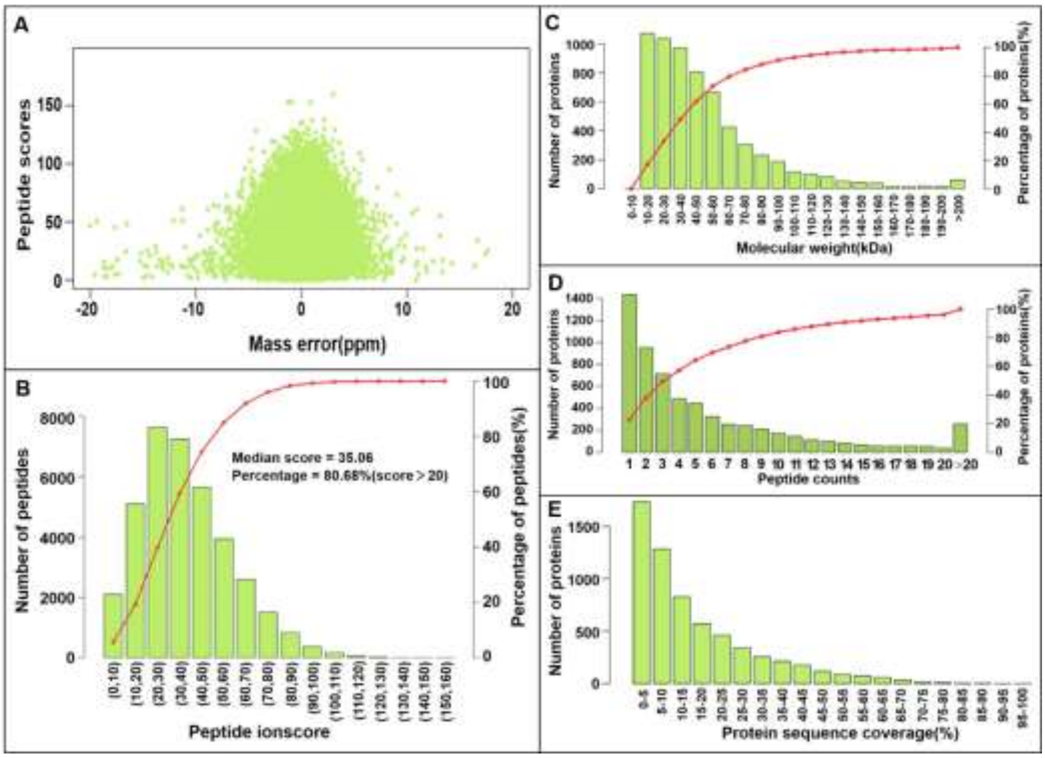

Figure S1. Quality control validation of MS date. (**A**)Mass error distribution; (**B**)Peptide ionScore distribution; (**C**) Protein molecular weight distribution; (**D**) Peptide count distribution; (**E**) Protein sequence coverage distribution.

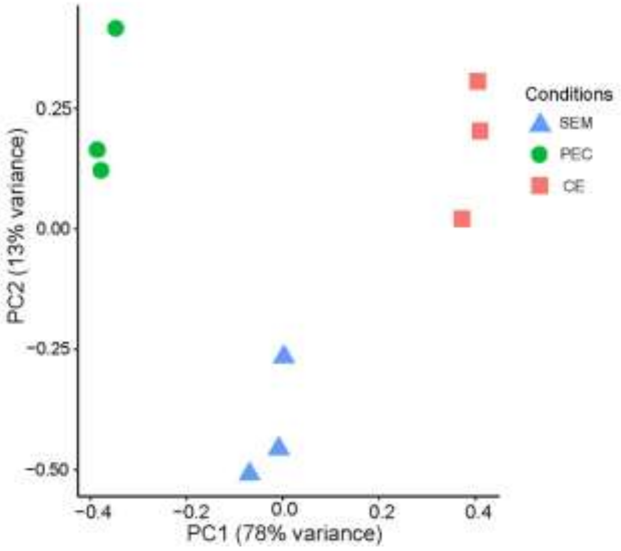

Figure S2. PCA plots from the whole proteomics of three stages of SE.

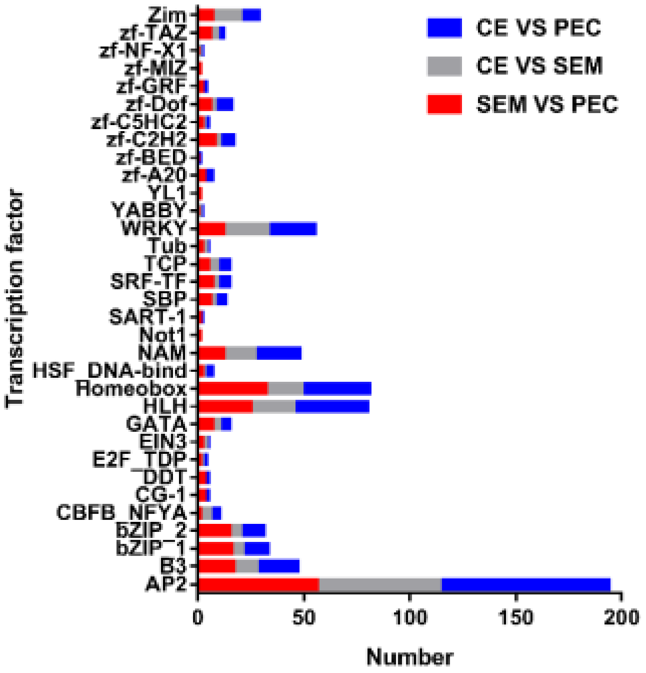

Figure S3. TFs identified in different stages of SE in *L. olgensis*.
